# Technologies for an inclusive robotics education

**DOI:** 10.12688/openreseurope.13321.2

**Published:** 2021-06-18

**Authors:** Dimitris ALIMISIS

**Affiliations:** 1 EDUMOTIVA (European Lab for Educational Technology), Kapnikareas 19A, Athens, 10556, Greece

**Keywords:** learning technologies, robotics education, maker movement, inclusive education, INBOTS.

## Abstract

The H2020 project “INBOTS: Inclusive Robotics for a Better Society” (2018­–21) has worked in different disciplines involved in the acceptance and uptake of interactive robotics, including the promotion of accessible and multidisciplinary education programs. In INBOTS, educational robotics is considered as a learning tool that can bring robotics into school classrooms and benefit all children regardless of their future educational or professional orientation. Aiming to make robotics education inclusive, INBOTS has introduced a paradigm shift inspired by sound pedagogies (Papert’s constructionism) and emerging educational trends (the maker movement) and focused on creativity and other 21 ^st^-century skills. However, the realisation of this new paradigm requires appropriate curricula and technologies at both hardware and software levels. This paper addresses several questions and dilemmas related to the technologies currently in use in robotics education and the kind of technologies that can best support the proposed paradigm. This discussion results in specific criteria that robotics technologies must fulfil to foster the new paradigm. Based on these criteria, we review some representative technologies in both hardware and software. Then, we identify and discuss some technological solutions that exemplify the kind of technologies that can best support inclusive robotics education and make the proposed paradigm feasible. Finally, we show how some of these technologies can be combined to design a creative and inclusive project consistent with the criteria set in this paper.

## Introduction: a new paradigm in educational robotics

More than ever before, educators are using robotics kits and educational robots to teach and inspire students of all ages, from pre-school to secondary school, preparing them for a society in which robotics are used more and more often in everyday life. Robotics education is usually conceived as a two-fold mission: education *in* robotics and education *with* robotics. The *in* case promotes knowledge and understanding of the design, analysis, application, and operation of robots; the *with* concept is broader and entails the use of robotics as a tool for teaching and learning science, technology, engineering, arts and math (STEAM) subjects and developing 21st-century skills: creativity, problem-solving, critical thinking and teamwork, for example 1, 2.

This distinction between the two branches of robotics education is important because it affects the inclusiveness of the project. Learners in school education are still shaping their interests in and attitudes to different school disciplines, including engineering, technology, and robotics. Only a few of them will pursue university studies or professional careers in robotics or engineering or become software developers. However, education *with* robotics promises benefits for all children, no matter their future educational or professional orientation ^1^. Is this great promise and potential of robotics education considered and respected in the design of new curricula and technologies before they are brought into classrooms?

The H2020 project “INBOTS: Inclusive Robotics for a Better Society” brings together experts from different disciplines involved in the acceptance and uptake of interactive robotics, including the promotion of accessible and multidisciplinary education programs. In INBOTS, educational robotics is considered as a learning tool that can bring robotics into school classrooms and benefit all children regardless of their future educational or professional orientation. The INBOTS education team has worked (2018–2021) to develop a sustainable framework that will promote inclusive robotics education at EU level, focusing on education with robotics for three age groups: pre-school (age 4–6), primary school (age 7–12) and secondary school (age 13–17).

The concept of inclusive education in the INBOTS framework is clearly distinct from accessibility for children with disabilities ^3^. In this paper, inclusiveness relates to the broader social uptake of robotics ^4^ and refers to an education that will make the multiple benefits coming from robotics education accessible for all the children, demystify robots and promote the future adoption of robots in their careers and everyday life. This concept of inclusiveness comes to challenge the misconception that fluency with robotic technologies is one more vocational skill relevant only for students who will follow robotics or engineering careers in the future. This misconception, coupled with gender-biased views that robotics is a “male” subject, may result in discouragement or exclusion of students not interested in engineering careers and especially the female ones ^5^. The wide spread of robotic technologies in everyday life and more importantly the wide range of skills that can be developed through robotics education, including the so-called 21st century skills, dictate a shift from discriminatory misconceptions to the recognition that robotics education can offer knowledge and skills valuable for every citizen. Such a shift requires a radical change in the current core concepts and practices of robotics education that will result in the transformation of robotics education towards a new paradigm inclusive of all children.

We have already outlined in another paper ^5^ an inclusive paradigm that is inspired by sound pedagogies (Papert’s constructionism ^6^) and emerging educational trends (the maker movement in education ^7, 8^). The new paradigm integrates the maker culture ^9^ and the do-it-yourself (DIY) ethos in robotics education with a focus on creativity and the other 21st century skills: problem-solving, critical thinking and teamwork. During the last decade, constructionism and maker movement have already been introduced in educational contexts. According to a systematic review ^10^ most of the existing research studies in robotics education have used constructivist and constructionist frameworks to design and implement robotics curricula and to analyse young children’s engagement in robotics education. However, the dominant framework seems to be different when it comes to the several curricula provided by the producers of popular commercial robotic kits which dictate the use of the kits by teachers and children and inevitably influence robotics education. A recent review ^11^ of some typical educational robotics curricula supporting well-known technologies has revealed a chaotic landscape where curricula rarely share common methods and principles. Their development does not seem to follow a generic and comprehensive model. Most of those curricula propose usually a restricted step-by-step guided approach for learners or strictly scripted workshops to assembly and program prescribed models of robots that do not lead to any meaningful learning ^12^. This approach is far away from a constructionist or maker education framework and cannot foster creativity, inventiveness, and curiosity for children ^13^.

In the INBOTS framework, we are aware that the realisation of a new paradigm in education depends on appropriate curricula and technologies. We have already reviewed the more prominent curricula and recommended changes to align them with the proposed paradigm ^11^. In addition, a set of specific curricula and resources for school education that exemplify the new paradigm have been developed. The INBOTS curricula and resources have been piloted with teachers and children in courses in Athens (Autumn–Winter 2019); a short video from the pilots is available on YouTube. The curricula and resources are available online to help teachers and educators implement the proposed paradigm in their classes and labs and inspire them to create their own curricula and resources.

The new paradigm – and the INBOTS curricula – needs support from appropriate technological tools. We have already provided a systematic review of the available educational robotics technologies that appear in the literature ^13^ and a list of available resources.

Following up on this work within INBOTS, the present paper explores a central question emerging from our previous work: what kind of technologies (at hardware and software levels) are appropriate to support the new paradigm and engage young people with an inclusive robotics education? To answer this question, we need first to answer the following sub-questions:

– What criteria should inform decisions about the potential of particular technologies to support an inclusive robotics education?

– To what extent can the technologies currently used support an inclusive robotics education?

The following section addresses these questions by examining some critical dilemmas regarding technologies used in robotics education. In the process, we identify criteria that robotics technologies must fulfil to foster the new paradigm. We subsequently use these criteria to review some representative technologies at both hardware and software levels. We then propose and discuss some exemplary technological solutions to demonstrate the kinds of technologies that support inclusive robotics education and make the proposed paradigm feasible. Finally, we show how some of these technologies can be combined to design a creative and inclusive project compatible with our paradigm.

## Discussion

Before exploring the technologies currently used in robotics education and how well they serve our proposed paradigm, it is necessary to establish some criteria for this discussion. To this end, some important questions and dilemmas within robotics education are highlighted in the following sub-sections.

### Establishing criteria

*Arts and crafts robots or LEGO ^®^ MINDSTORMS robots?* ^14^. This challenging dilemma comes from the title of a comparative study ^14^ in which students in one group were asked to build a robot from scratch using craft and recycled materials while those in a second group were asked to build a robot out of structured materials. Results showed that building a robot from scratch increased pupils’ knowledge and manual skills while building a robot with structured materials increased their awareness of the robotisation of machines.

In other papers ^4, 15^ we have reported upon a project realised in the frame of the eCraft2Learn action in which children aged 13–17 created DIY robotic automobiles from scratch using low-cost and recycled materials; this project aligns with the “make your own robots” concept and the maker movement mindset. In the frame of the same action, we have seen young students in several projects working enthusiastically with creative “arts and crafts” robotic artefacts ^15^.

We concur with researchers who argue for technologies such as “creative material” ^16, 17^ that can offer a design which provides hands-on material for children to manipulate, think with and act upon as creators. The “arts and crafts” model ^14^ allows children to un-box, un-craft, deconstruct and reconstruct, triggering their curiosity and facilitating collaboration; it ensures transparency and visibility of tools and materials and helps children understand how their robot works, its boundaries and its uses ^16^.

*Technologies designed for professionals and hobbyists or for learners?* Though the answer to this question seems (and is) obvious from a learning perspective, it is a common scenario in robotics education for teachers and learners to struggle managing technologies designed mainly for other purposes and only secondarily for education, if at all. For instance, prototyping technologies designed for professionals or enthusiasts, such as Arduino-like microcontrollers, can, when introduced in classrooms ^4^ or makerspaces for novices, cause difficulties and eventually frustration and discouragement because they presuppose a domain knowledge (electronics) and skills (such as soldering, circuiting, C coding etc.) that young students or novices are unlikely to possess. Moreover, the producers of technologies for professionals or hobbyists focus on doing the job fast and at a low cost. They do not care much (nor is it their role) to help users understand how the product works; this often results in “overdesigned” technologies which essentially hide how the product actually works.

We have already argued ^1, 5^ that the technologies we invite children to interact with should be designed in line with sound learning theories and constructionist/constructivist pedagogy ^6, 18^. However, this premise alone cannot guarantee the design of appropriate tools. Very often, technologies for learning offer “less for more” compared to corresponding professional tools or are not reliable. Putting emphasis on making technologies educationally meaningful and engaging should not compromise their reliability or scientific accuracy.

*Should technologies prepare students for engineering jobs or develop personal expression and skills?* Decisions regarding which objectives to pursue in robotics education have evident consequences for the choice of technologies deployed ^12^. If the emphasis is on preparing students for engineering jobs, then technologies replicating or simulating professional tools may be the right choice; however, if we focus on personal expression and skills development for all, regardless of future careers, then technologies compatible with the above-mentioned “arts and crafts” model, which allow children to design and make their own robotic artefacts from scratch, seem the right choice.

*Should more time be spent on technicalities or creative tasks?* When students start working on their robotics projects, they need some initial training to learn to use the technology. The time required for this training is an important factor for student engagement. According to our experiences in pilots ^4^, children are impatient to see concrete results and feel frustrated or even disappointed when they encounter difficulties using the tools or when it takes too long to progress from technicalities to creative tasks. Moreover, the attention demanded by technical issues may compromise their interest and undermine their engagement in the project. Thus, to allot more project time to creative tasks and less to technicalities, we need technology that is easy for learners to understand and use without exposing them to an unnecessary level of technical detail ^12^.

*Scenarios from everyday life or “missions to Mars”?* Very often in robotics competitions (e.g. the World Robot Olympiad) or in the curricula and manuals proposed by the producers of commercial robotics kits, “exotic” scenarios and models of robots inspired by fiction movies or popular TV shows are proposed to excite children’s interest. Industries advertise spectacular robots ready for space adventures, which sound exciting but have nothing to do with children’s everyday lives. There is a risk that this sort of marketing will result in the mystification of robots and promote misconceptions about how robotics technologies work and the reasoning behind them ^5^.

*Cost matters.* Though less directly connected to learning and cognition ^12^, the high cost of robotic technologies may be an inhibiting factor for schools, educators and families ^19^. Our experiences from the eCraft2Learn Project ^4^ have shown that. low-cost kits combined with materials from everyday life and recycled components of toys can offer creative solutions and higher educational value than expensive, ready-made robots or kits that allow children to make only a limited number of predefined models.

From this discussion the following main criteria arise for the kind of technologies that can serve best our inclusive paradigm:

– enable students to build DIY robots from scratch.

– offer transparency of their underlying structure.

– are designed for learners and not for professionals or hobbyists.

– do not require prior knowledge of the technology from outside the classroom.

– emphasise personal expression and skills development for all instead of preparing students for engineering jobs.

– allow for more time to be spent on creative tasks and less on technicalities.

– demystify robots and help children understand how their robot works.

– are of low cost for schools.

How do the technologies currently in use perform against the criteria set in the previous section? In Table 1, three popular platforms (LEGO Mindstorms, Arduino and Micro:bit) are assessed against these criteria in an ordinal scale consisting of a spectrum of 5 descriptive qualities: excellent, good, average, poor, very poor. This qualitative comparison is aimed to inform a discussion of the kinds of technologies that might best serve the proposed paradigm.

**Table 1.** Comparing the performance of platforms popular in educational robotics against the criteria arising from the discussion at an ordinal scale: excellent, good, average, poor, very poor.

Criterion	LEGO Mindstorms	Arduino	Micro:bit
enables students to build DIY robots from scratch.	poor	excellent	excellent
offers transparency of their underlying structure.	poor	excellent	excellent
is designed for learners and not for professionals or hobbyists.	excellent	poor	good
requires prior knowledge of the technology from outside the classroom.	excellent	very poor	good
emphasises personal expression and skills development for all instead of preparing students for engineering jobs.	good	average	excellent
allows for more time to be spent on creative tasks and less on technicalities.	excellent	poor	good
demystify robots and helps children understand how their robot works.	good	excellent	excellent
is of low cost for schools.	very poor	excellent	excellent

The three platforms compared in Table 1 were selected because they are among the most popular ^13^ and represent quite different approaches within educational robotics. Moreover, they have attracted considerable interest by the research community and have been subject of numerous studies exploring their educational potential and shortcomings (e.g. 20– 22). LEGO Mindstorms offers modular assembly of robots based on the popular LEGO construction sets for creative building. The Arduino platform, like many other microcontrollers available for physical computing, is based on boards that can read an input and turn it into an output, activating a motor or turning on an LED; teachers and students use these to build simple or complex robotic artefacts at low cost. The BBC Micro:bit platform offers an accessible pathway into electronics and computing, combining ease of construction with the power of engineering. Educators and children can use sensors and actuators connected to their Micro:bit board to make robots and other digital artefacts.

As Table 1 shows, LEGO Mindstorms and Arduino platforms meet some, but not all, of the set criteria, while Micro:bit performs quite well in most categories. The popular commercial platform LEGO Mindstorms builds on the familiarity to children with LEGO bricks and offers (though at high cost) easy modular assembling of robots and abstraction of unnecessary technical details. However, it lags behind the other two platforms in terms of openness to manipulation by the learner, transparency of the underlying electronics and potential to become a design material.

Arduino platform, though designed primarily for professionals and hobbyists, offers more opportunities for learners to “make their own robots”, can easily become a design tool in the hands of learners, and provides many opportunities for learners’ own manipulations. However, Arduino requires prior knowledge of the technology from outside the classroom (e.g. electronics, soldering skills). Moreover, students usually have to spend more time on technicalities than with LEGO Mindstorms or Micro:bit and may be frustrated by the many unnecessary details to which they are expected to attend. We concur with Blikstein: “a toolkit that is supposed to help students learn robotics but makes them spend half of their time figuring out how a breadboard works, will not be able to live up to its goals” ^12^.

The Micro:bit platform seems to excel in certain aspects when compared with LEGO Mindstorms and Arduino. It carries most of the advantages of Arduino without some of its shortcomings. The electronics in Micro:bit are comparatively more transparent, without exposing users to unnecessary details; less prior technical knowledge and skill is required. Micro:bit combines expressive electronics with arts and crafts, and it is a good example of an inclusive technology that enables children to make their own low-cost robotic artefacts to support inclusive education in robotics, in line with our discussion in this article.

Of course, some of the criteria in Table 1 can be influenced at some extent by the teacher’s intervention. For instance, regarding the performance of Lego Mindstorms against the 1st criterion (“enables students to build DIY robots”), an aware teacher may introduce in the class some crafting that will enable students to decorate their Lego robot which is in line with the DIY criterion but not equivalent to making a robot from scratch; or regarding the 5th criterion (“emphasises personal expression and skills development for all …”), an aware teacher again can encourage students to combine Arduino with appropriate shields or other kits (see solutions we propose in the last section of this discussion) to improve its potential for personal expression and skills development. The role of the teacher is always important; however, the design decisions and principles of each platform imply (or impose) certain learning pathways for users with consequent benefits or shortcomings like those depicted in Table 1.

### Reviewing programming languages and tools

The programming language used is another factor that differentiates robotics technologies. Both text coding and visual programming languages (VPLs) are used in educational robotics. Text coding (e. g. Arduino IDE) is synonymous with a technical language and a rigorous syntax, while VPLs offer commands in visual blocks which prevent syntax errors. Some programming platforms (e.g. MakeCode) incorporate both options: children can use programming blocks in a visual environment while watching their commands appear in a second window in Python or JavaScript, which familiarises them with text coding.

In Table 2, Table 3 and Table 4, three different programming solutions for each of three common tasks are listed and then discussed. These tasks (turn on the LED in Table 2, play a note in Table 3, rotate the servo motor in Table 4) are ones that might be involved in classic projects in which children make a lighthouse, a Theremin music box and a sunflower, respectively. The (partial) solutions come from programming platforms well known in educational robotics: Arduino IDE (as an example of text coding with commands written in C/C++ language) and two VPL platforms ( MBlock and Open Roberta Lab) that use visual blocks instead of text commands.

**Table 2.** Comparison of three tools for programming the task “turn on the LED”.

Programming language	Lighthouse project/task: turn on the LED
Arduino C	digitalWrite(9, HIGH);
Mblock	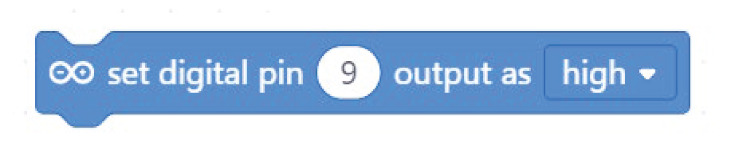
Open Roberta Lab	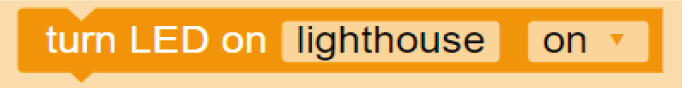

**Table 3.** Comparison of three tools for programming the task “play note”.

Programming language	Theremin project/task: play note
Arduino C	tone(buzzer_buzzer, 300, 100)
Mblock	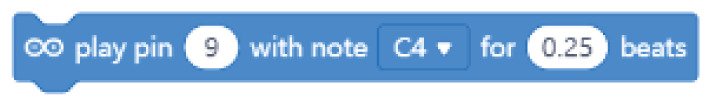
Open Roberta Lab	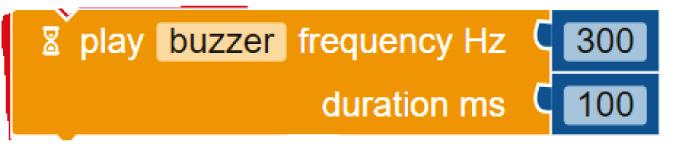

**Table 4.** Comparison of three tools for programming the task “rotate the servo motor”.

Programming language	Sunflower project/task: rotate the servo motor
Arduino C	servo_Sunflower.write(90)
Mblock	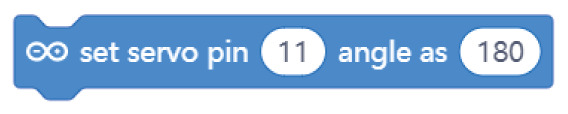
Open Roberta Lab	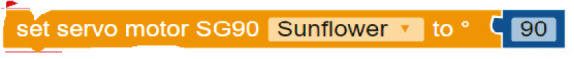

Comparing the text coding and block-based solutions in Table 2, Table 3 and Table 4, we can first observe that the technical language used by Arduino C is not the most practical for young students, who are not familiar with technical languages. For instance, the students might not understand terms such as “digitalWrite” or “HIGH” or “LOW” ^12^. The same issue arises with the MBlock editor, which, although it uses visual blocks to change the text commands, still uses the same terminology.

In contrast to Arduino C and MBlock, Open Roberta Lab uses more widely recognised terms (“ON-OFF”, for example, instead of “HIGH-LOW”) and, in general, uses terminology that makes more sense for children. For instance, instead of “digitalWrite” or “set digital pin”, it speaks a more human language: “turn LED on” or “play buzzer” with particular “frequency” and “duration”.

These examples show that, if we wish to provide learners with a more intuitive and meaningful programming interface, it is not enough to move from text coding to visual blocks; we need, at the same time, to rethink the role of a technical language that is well established with professionals and hobbyists but may be strange for young learners outside these communities. This reasoning applies not only to text coding but also to the terminology used inside the visual blocks which in many cases replicate the technical terminology used in text coding. The aim should be to use a more “human” language that is meaningful for young learners and helps lower the floor for children’s participation.

However, it is important to do so in ways that do not also lower the ceiling for what children can learn and build with them ^23^. With this in mind, we find valuable for learners the opportunity to have parallel access to visual blocks and text coding tools. For instance, when learners program their Arduino board using visual blocks in Open Roberta Lab, the Arduino C text code is automatically generated and appears in a second window. This parallel access is intended to familiarise children with text coding and prepare them to jump into a more advanced programming tool when they are ready for it.

Another problem we identify in the Arduino C commands ( Table 2, Table 3 and Table 4) is the “relatively unforgiving” ^24^ and rigorous syntax required. This poses unnecessary obstacles for young programmers, who must pay attention to details of syntax and manage frequent syntax errors. This issue relates to the discussion about the role of “boring” technicalities, such as difficult syntax and unfamiliar terms in the programming interface that students must use to create behaviours for their robots. In contrast, visual programming solutions relieve children from worries about syntax and replace technical terms with a more intuitive and familiar language ^25^.

### Reviewing more technologies that can support the inclusive paradigm

 The maker movement has inspired a range of efforts to produce STEAM and robotics kits and toys. In this section, some indicative examples are reviewed to discover how well they may serve our paradigm for robotics education based on the analysis of Table 1, Table 2, Table 3 and Table 4. The review begins with technologies that largely target younger (primary school) learners and continues to those mainly intended for older learners (secondary school).

**Figure 1.**
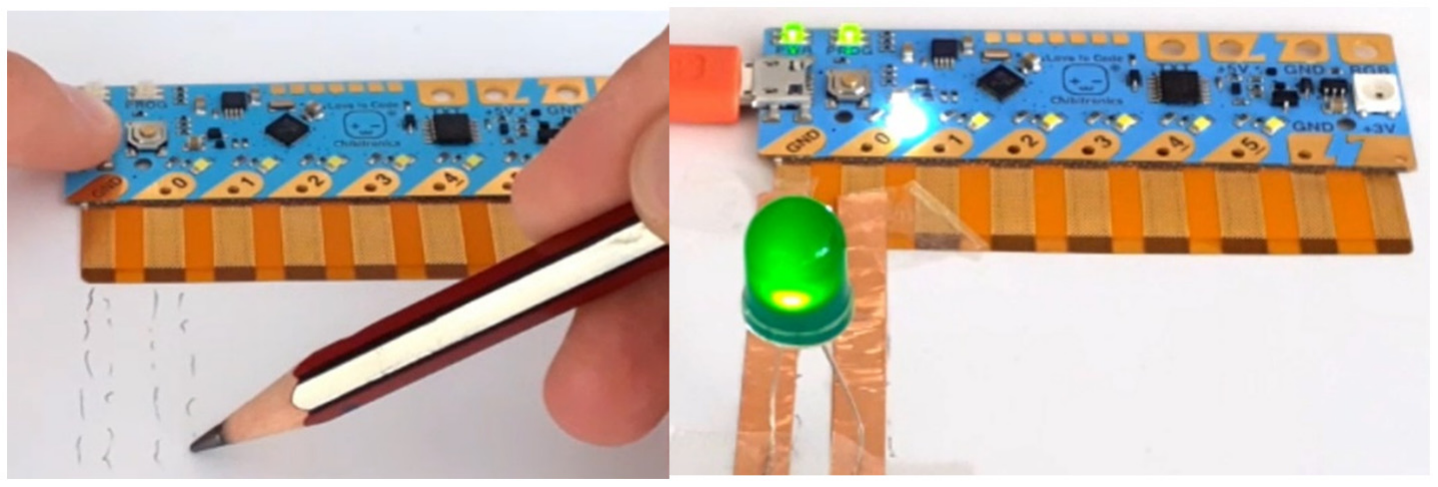
Making a simple LED circuit with Chibi Chip board and strips of copper tape as conductive material.

**Figure 2.**
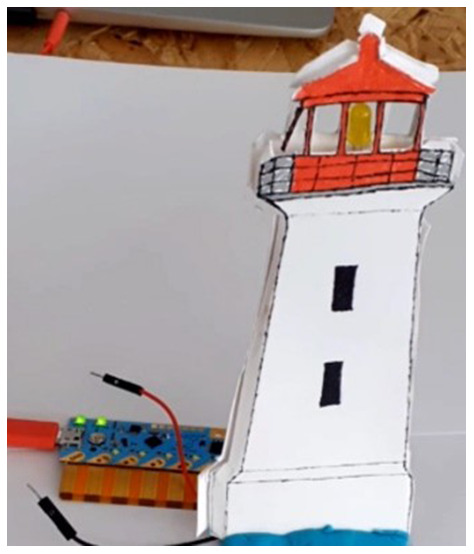
The lighthouse project with Chibi Chip board, an LED circuit and some crafting.

**Figure 3.**
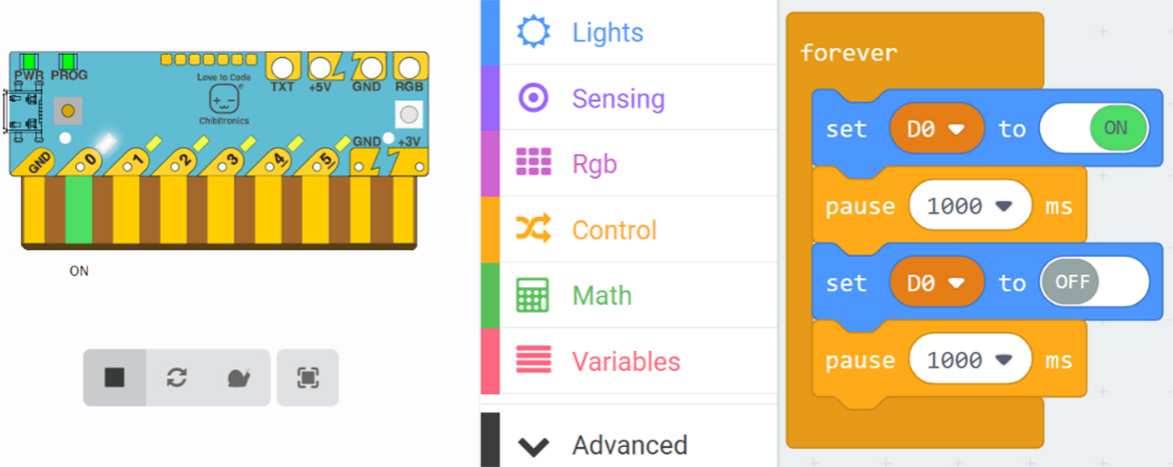
Simulation of the Chibi Chip board (left) and the code for the lighthouse blinking with MakeCode (right).

Cardboard and paperboard-based crafting have become a nearly ubiquitous entry point into making and “can provide an easily accessible pathway into electronics and computing combining two modes of making, the power of engineering with the expressiveness of craft” ^26^. Chibitronics offers a friendly way for kids to learn, design and create their own electronics projects using paper circuits ( Figure 1), arts and crafts ( Figure 2) and friendly programming ( Figure 3). Children can use the Chibi Chip board ( Figure 1) to bring their projects to life by connecting switches and sensors to it. The materials used look like familiar craft supplies; for instance, strips of copper tape or other conductive material are used to connect the Chibi Chip board to the LED (or other) circuit in a loop ( Figure 2). The focus in Chibitronics is on combining expressive electronics with arts and crafts, and it currently supports only a few sensors and actuators, which means fewer options for robotics projects than Arduino-like microcontrollers can offer. However, this is a good example of a technology that enables children to make simple, low-cost arts and crafts projects, in line with our discussion.

Table 1 shows that different technologies come with different advantages and shortcomings. For instance, we have noted that LEGO Mindstorms abstracts unnecessary details, limits the time spent on technicalities and is familiar to children; however, it also has only a low potential of becoming a design material and carries a high cost. On the other hand, Arduino offers, at low cost, a high potential of becoming a design material, but it does not abstract unnecessary details and requires considerable attention to technicalities.

With these remarks in mind, we welcome efforts to combine different technologies, taking the strengths of each kit and not its weaknesses. For instance, imagine a solution that would combine the abstraction of LEGO Mindstorms with the power and flexibility of RaspberryPi or Arduino. Indeed, such solutions have already been proposed and entered the market. For instance, the BrickPi board and the PiStorms board, among others, replace the LEGO NXT/EV3 brick with the Raspberry Pi or Arduino board and offer an extension that makes it compatible with the LEGO Mindstorms platform. For this purpose, a “shield” is added to the Raspberry Pi or Arduino board that offers connectors to the LEGO Mindstorms sensors and motors. Combining the processing power of the low-cost Raspberry Pi or Arduino board with the convenience of the LEGO motors, sensors and building system results in a better tool than either system offers alone.

**Figure 4.**
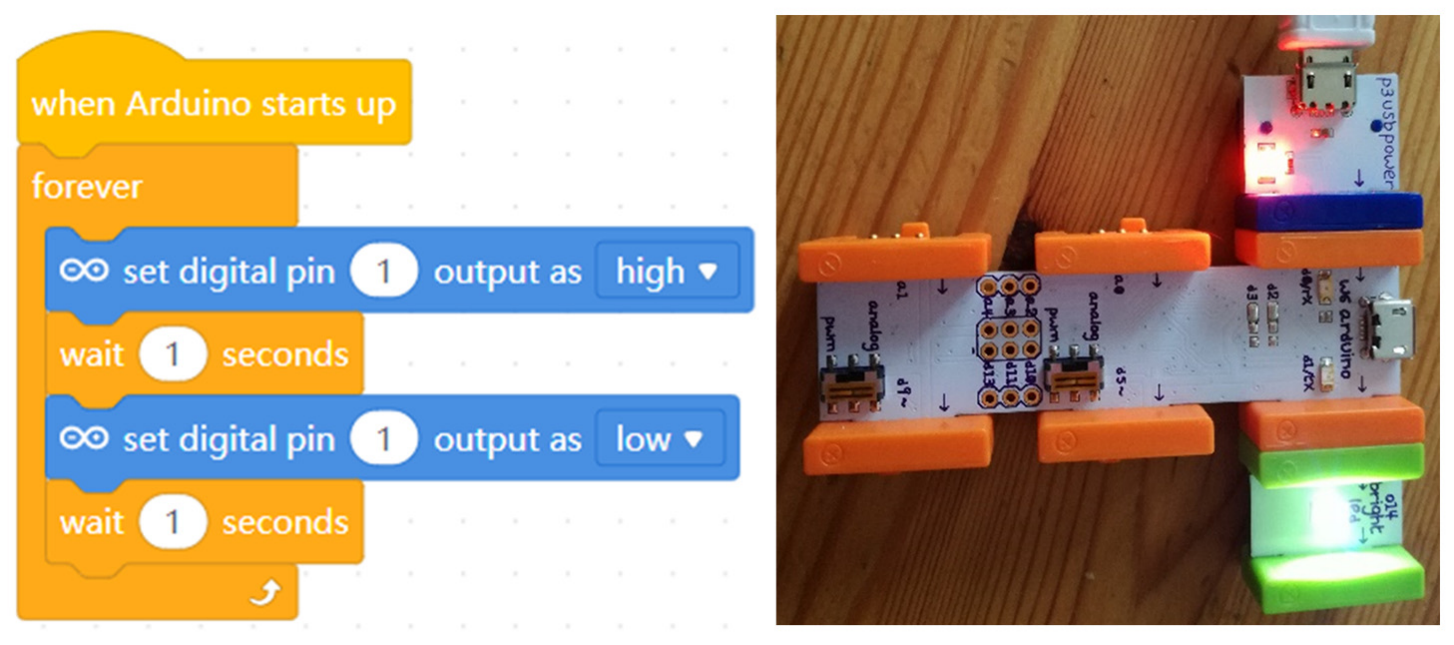
The common blink project with littleBits CodeBit (right) and MakeBlock code (left).

In another example, the well-known littleBits series offers the littleBits Arduino Bit, a microcontroller that gives the power to code the littleBits circuits and control the way lights, buttons, motors, sensors and other Bits interact. The Arduino Bit can offer the functionality of an Arduino Leonardo without any breadboarding, soldering or wiring. It is simply plugged into a computer, snapped together with other Little Bits and programmed with the visual blocks–based Code Kit App or with MakeBlock ( Figure 4).

Very often, a breadboard is used in connection with a microcontroller to make electric circuitry. Our experiences in our pilots with young learners have shown that difficulties and misunderstandings arise for these students while working with a breadboard; they do not understand how its holes are connected internally or why the breadboard differs from the standard diagrams used in physics lessons. Can we improve the design of the breadboard to make it friendlier and more meaningful for young learners?

In an interesting project aimed at strengthening the understanding of the breadboard and closing the gap between its representation in the diagram and the physical breadboard, a 3D-printed plastic cover was designed as an overlay to fit on top of a standard breadboard. The cover visualised the internal connections between the holes, and the supply lines were coloured red and black. In addition, a new type of diagram was designed to complement the new type of breadboard and to further explain the components and connections in comparison with a standard electrical diagram ^27^. In the eCraft2Learn pilots ^15^, we found that working with a simulation of the breadboard in Tinkercad Circuits software before going to a real breadboard helped young learners understand how to use the breadboard in real circuits.

Several efforts and solutions have focused on adapting the popular Arduino-like microcontrollers to make them friendlier and more manageable for young learners, especially novices in electronics and coding. One popular solution is the use of a “shield”: a piece of hardware that can be mounted on a microcontroller to give it a specific purpose or extra capabilities. For example, a motor shield makes it easier to control motors with a microcontroller, and an Ethernet shield connects it to the Internet.

There are many different shields for a wide variety of purposes. The Arduino Education Shield is a custom-made shield designed by Arduino Education, specially tailored for educational purposes to enable quick and easy learning while building projects and to make connecting and prototyping easier through a simplified design. It connects push button modules, light sensor modules, power LED modules and more to an Arduino board and extends its capabilities, making the creation of projects easier.

Using shields instead of the corresponding circuitry and wiring to the microcontroller board offers many advantages. Learners do not need to worry about the circuitry since all the components they need are on the shield. They can easily detach the shield from the microcontroller board and reattach it whenever they want, without worrying about making the circuit and wiring everything again. Using shields leads to fewer errors than the process of connecting the separated parts. Finally, shields offer an easy way to add new features to an Arduino-like board that otherwise would be difficult to create.

Grove by Seeed Studio is another interesting solution that provides an open, modular system designed for easy connection of sensors or actuators to a microprocessor, thus making it easy to connect, experiment and simplify the prototyping process without wiring or soldering. Recently, Arduino and Seeed Studio announced the Arduino Sensor Kit – Base: a plug and play addition to the Arduino board to get users started with electronics and programming. The kit helps to connect and program basic Grove modules that include both sensors and actuators – again without requiring a breadboard, soldering or wiring. Children can also use the compact and flexible Proto Shield kit to build their own Arduino Shield. The Proto Shield kit makes it easy to design custom circuits and solder electronics directly on it.

A simplified type of shield is Snapino, which is helpful for beginners and novices and offers a useful introduction to the Arduino board. Snap Circuits use electronic blocks that snap onto a clear plastic grid to build different circuits. The Snapino module is an Arduino UNO microcontroller mounted on a Snap Circuits base. When combined with Snap Circuits, which has electronic parts and modules mounted on snaps, it offers a simple and easy prototyping platform. Because LEDs come with internal resistors, users do not need to worry about protecting them. A base grid is provided instead of a breadboard for mounting parts and wires.

Summarising these reviews in relation to the criteria set in this discussion, we argue that several technologies are already available which, at a lower or higher degree, can support an inclusive robotics education paradigm inspired from the maker movement. The main innovations we have found useful to this end include: the combination of expressive electronics with arts and crafts (e.g. Chibitronics) that enables children to make simple, low-cost robotic artifacts from scratch and provides opportunities for personal expression; the combination of two different kits in one platform (e.g. BrickPi, PiStorms) that can result in a better tool taking the strengths of each kit and not its weaknesses; improvements in the design of breadboards (e.g 12) or use of simulations (e.g Tinkercad Circuits) that can help make breadboards friendlier and more meaningful for young learners; finally, shields used instead of the corresponding circuitry and wiring to the microcontroller board that offer a simple and easy prototyping platform and an easy way to add new features to the microcontroller board. While each of the reviewed technologies contributes to address specific issues related to the criteria set, they don’t provide an overall solution. However, these innovative solutions may offer valuable insights into the pathways that the design of educational robots could follow in the years to come.

### Combining some of the tools introduced above to design a robotic car

**Figure 5.**
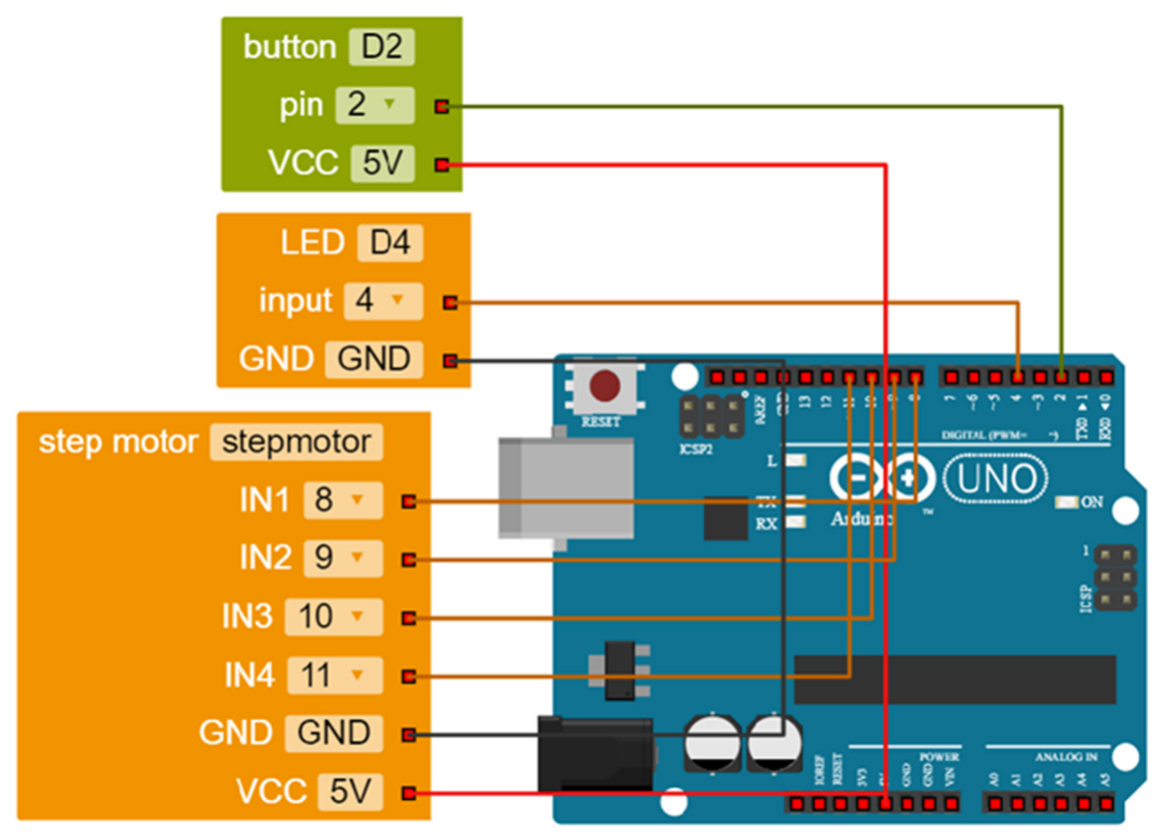
Robot configuration in Open Roberta Lab.

**Figure 6.**
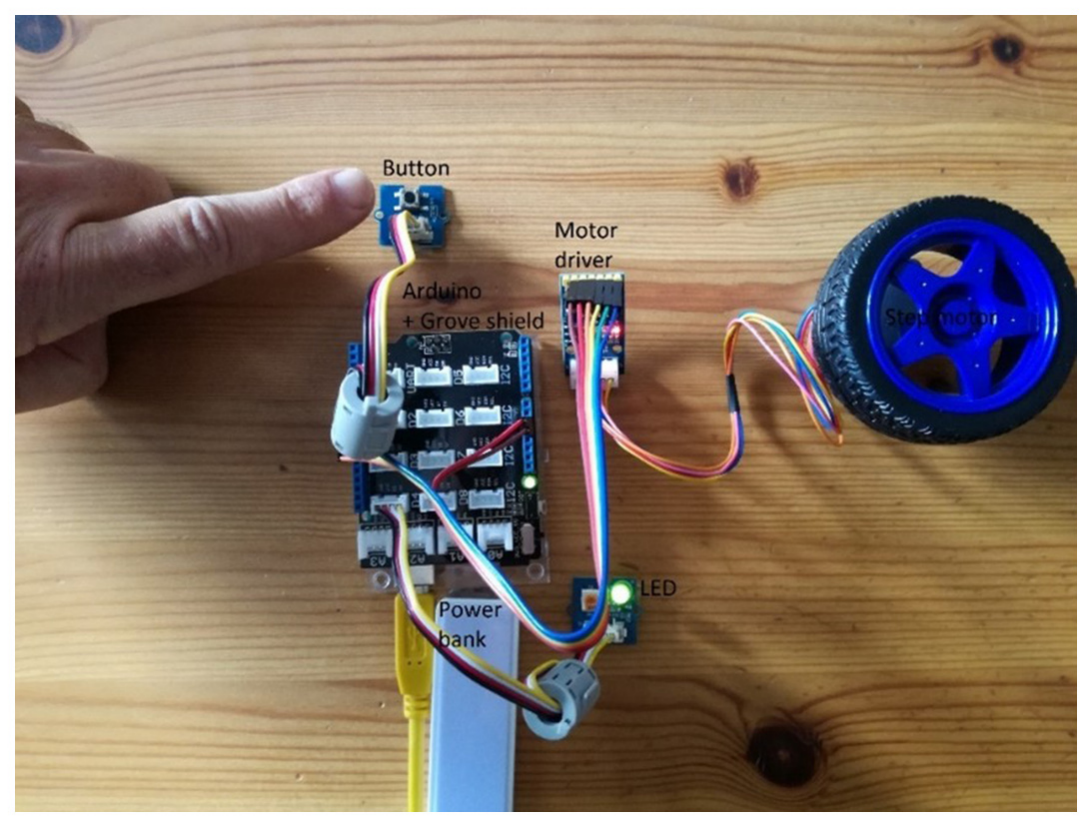
Combining Arduino board with Grove shield and modules to design a robotic car so that when a button is pressed its lights turn on and it moves forward.

To conclude the discussion, we present a common project in educational robotics using some of the tools introduced above to show how these can be combined to help young makers create easy, meaningful and realistic projects. The hypothetical project involves designing a robotic car. The task is to design it so that when it receives a signal from a sensor (as a result, for example, of a button being pressed) its lights turn on and it moves forward. In Figure 5, the robot configuration is illustrated as it was made in the Open Roberta Lab software. In Figure 6, the real design is demonstrated; a Grove shield is attached to an Arduino board where a button, an LED and a step motor driver module are snapped without any soldering, breadboard or wiring.

**Figure 7.**
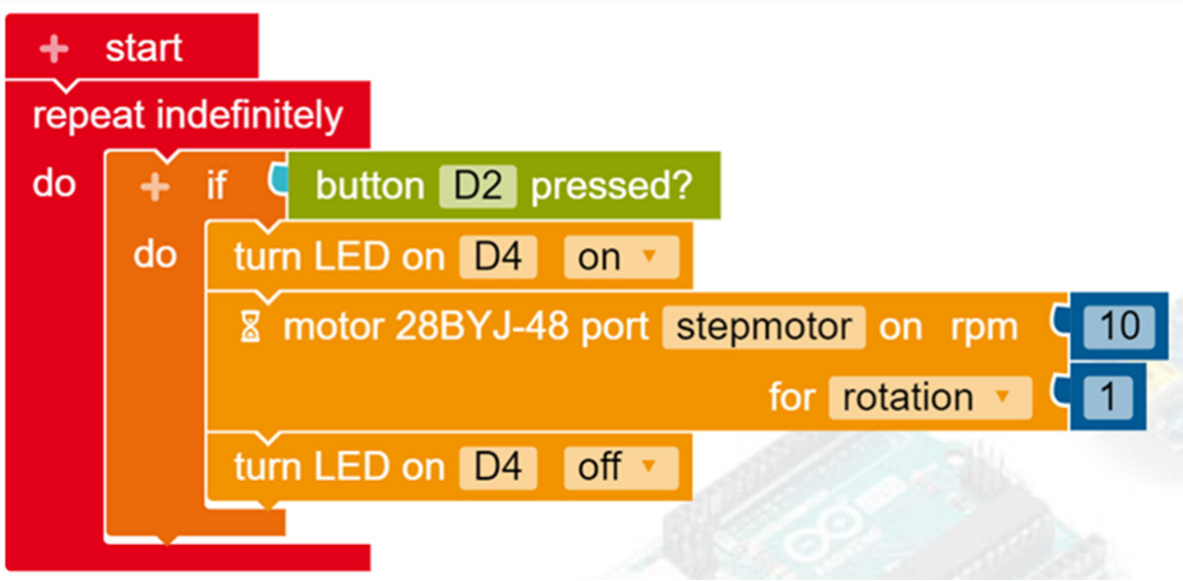
Programming the robot in Open Roberta Lab.

Figure 7 illustrates the code required to control the robotic car in Open Roberta Lab; coloured blocks are easily snapped together, preventing syntax errors. The visual form of the blocks (e.g. repeat indefinitely) aids understanding of the underlying concepts (e.g. loop). The terms in the blocks are remarkably close to normal usage, making the programming task meaningful for young learners. The blocks can easily be parameterised (e.g. rotations per minute, number of rotations) in a flexible way according to the requirements of the project. This is a basic design that learners can expand, adding more wheels and sensors, crafting a chassis or decorating their creation according to their own design.

## Conclusions

This paper explores technological solutions and tools that can contribute to a more inclusive robotics education. We review key dilemmas regarding the use of technologies in robotics education, offering a perspective that might be useful in both evaluating technologies currently in use and developing new tools and solutions to promote an inclusive robotics education. We criticise deficiencies and shortcomings of technologies currently popular in the field and emphasise that technologies brought into classrooms should be those designed for learners and not for professionals or hobbyists.

Technologies designed for professionals are usually difficult for young learners to understand and operate, and we must carefully consider their use in education in order to prevent a technocentric approach ^28^ in which learning is focused only on how to use the tool ^29^. Such a technocentric approach is precisely the opposite of the inclusive approach we adopt in this paper. We suggest several technological solutions and present an indicative scenario that aims at a didactic transformation of the “difficult” technologies before putting them in the hands of young learners. This didactic transformation involves the transformation of professional or scientific tools in a way that optimises comprehension and ease of use in the educational process.

Finally, this paper highlights the educational value of the “arts and crafts” model and the need for robotics kits that can become a hands-on design material for children to manipulate and think with. The paper provides examples of the kind of inventive and creative technologies we consider essential to an inclusive educational paradigm.

Further work is required to understand in more depth how technologies determine the models currently in use in robotics education and how designing new technologies or devising new ways to use existing technologies might help educational robotics communities to move away from technocentric models and towards an inclusive educational paradigm.

## Data and software availability

No data are associated with this article.
