# Supplementary material for: Technologies for an inclusive robotics education
Source: Open Res Eur. 2021 Jun 18;1:40. Originally published 2021 Apr 21. [Version 2] doi: 10.12688/openreseurope.13321.2 (PMC10445904; doi:10.12688/openreseurope.13321.2)
Supplement: Peer review history

# Peer review: Reviewer response for version 2

**DOI:** 10.21956/openreseurope.14892.r27836

**Published:** 2021-11-04

**Authors:** Pavel Petrovic

**Affiliations:** 1 Department of Applied Informatics, Faculty of Mathematics, Comenius University, Bratislava, Bratislava, Slovakia

This article formulates and discusses important questions that educators and their managers need to consider when selecting a technological didactical solution for applying electronics or robotics educational tools and approaches into educational process. It also provides an overview of current state of the art regarding the various technological solutions available. It tries to put into comparison a few of the notoriously used solutions highlighting their main strengths. The article would certainly be a recommended read for anyone considering implementation of robotics or electronics technology into their educational process, but it also serves as a useful resource with new ideas for some experienced users in the field.

 On the other hand, it certainly would not be the only reading resource recommended in such implementation scenario, since technology is only one (and less important) aspect of the story. Much more important would be WHICH pedagogical goals are going to be approached, WHAT is going to be done with the technology and HOW it is going to be used. Practically all of the technologies mentioned in the article are relevant in one or another educational scenario, and thus it is impossible to generalize over them at this level. Most of the time a disadvantage on one end gives some other advantage on another one, so the selection of the technology is very "task-dependent" issue and should be treated as such. Also, I would try to resist an urge or call for unifying the approaches, that the article suggested at some place, as diversity often helps evolution, creativity, and progress in general. For instance, I do not feel so happy about the current trend of all the educational environments for children have a tendency to look like Scratch. On one hand, yes, the child can move from one solution to another more easily and the learning curve for a new technology is faster. On the other hand, programming as such is quite at the crossroads still, and other approaches than the outdated von-Neumann-based control flow programming model need to be heavily investigated and put into use. For example data-flow programming, which allows natural parallelization while still keeping human-readability of the code, to name just one.

 I would also like to contest the positive assessment of the Micro:bit solution and its comparison to Arduino in Table 1. It certainly does not fall into the same price category and the versatility of Arduino is higher in several degrees of magnitude. The physical connector system of Micro:bit is a significant obstacle thus many of the workshops with Micro:bit end up using just a LED matrix display and the gyro - i.e. at a level of projects that could well be done on a computer, or let's say on a mobile phone or a tablet, if you like. However, without the extra cost, maintenance, storage, servicing, and all the other extra hassle with the hardware. Arduino platform IS specifically designed for learners contrary to the suggestion in the Table 1, and it does not require any more prior knowledge than Micro:bit. Ranging Arduino as poor in the category of creative time spending is definitely unfair too. It is just different, works different, and gives different set of experiences, which can sometimes be treated more valuable, because with Arduino, students learn about what happens inside. That matters. What value do you see in a child playing to be a wizzard who can impress others by legendary-kind of experiences: "Hey, LED, you should NOW TURN ON!" ...and then the LED turned on, and all the children remembered that till their last days and stayed happy forever. I know what you mean, but my point is that this comparison mixes uncomparable. They are all valid, very useful, and beautiful technologies made for a different purpose, that's it.

Are all factual statements correct and adequately supported by citations?

Partly

Is the review written in accessible language?

Yes

Are the conclusions drawn appropriate in the context of the current research literature?

Yes

Is the topic of the review discussed comprehensively in the context of the current literature?

Yes

Reviewer Expertise:

Educational Robotics, Artificial Intelligence

I confirm that I have read this submission and believe that I have an appropriate level of expertise to confirm that it is of an acceptable scientific standard.

# Peer review: Reviewer response for version 2

**DOI:** 10.21956/openreseurope.14892.r27137

**Published:** 2021-10-25

**Authors:** Amy Eguchi

**Affiliations:** 1 Department of Education Studies, University of California, San Diego, San Diego, CA, USA

Thank you for the revisions and responding to my comments. Regarding the new paradigm, maybe making the sub-title clearer will help readers understand your intention.

 The current title is "Introduction: a new paradigm in educational robotics". Instead, maybe you can say "a new paradigm in educational robotics for inclusive approaches". This way, it is clear that the new paradigm is not for educational robotics but to provide an inclusive learning experience for the learners.

 On page 6, the top of the left column, I would change the sentence to:

 "Of course, some of the criteria in Table 1 can be influenced at some extent by the pedagogical options that a teacher makes. For instance,

 regarding the performance of Lego Mindstorms against the 1st criterion (“enables students to build DIY robots”), a teacher who is familiar with how to integrate maker approaches in her/his teaching may ...."

Are all factual statements correct and adequately supported by citations?

Partly

Is the review written in accessible language?

Yes

Are the conclusions drawn appropriate in the context of the current research literature?

Yes

Is the topic of the review discussed comprehensively in the context of the current literature?

Partly

Reviewer Expertise:

Educational robotics, STEM education, Computer Science Education, curriculum development, AI literacy.

I confirm that I have read this submission and believe that I have an appropriate level of expertise to confirm that it is of an acceptable scientific standard.

# Peer review: Reviewer response for version 2

**DOI:** 10.21956/openreseurope.14892.r27138

**Published:** 2021-08-13

**Authors:** Sarah Matthews

**Affiliations:** 1 Faculty of Engineering, Architecture and Information Technology, The University of Queensland, Brisbane, Australia

This version has answered my pressing concerns, no further revisions are required.

Are all factual statements correct and adequately supported by citations?

Partly

Is the review written in accessible language?

Yes

Are the conclusions drawn appropriate in the context of the current research literature?

Yes

Is the topic of the review discussed comprehensively in the context of the current literature?

Partly

Reviewer Expertise:

Interaction design, educational technologies, creative materials for children, analysis of children's interactions with technologies.

I confirm that I have read this submission and believe that I have an appropriate level of expertise to confirm that it is of an acceptable scientific standard.

# Peer review: Reviewer response for version 1

**DOI:** 10.21956/openreseurope.14391.r26783

**Published:** 2021-06-01

**Authors:** Amy Eguchi

**Affiliations:** 1 Department of Education Studies, University of California, San Diego, San Diego, CA, USA

The paper presents an evaluation of popular robotics tools available for educators to use in the classroom as part of a larger project called "INBOTS: Inclusive Robotics for a Better Society". The evaluation focused on how the tool can promote learning opportunities for all students inclusively. The author came up with a set of eight criteria to assess the inclusiveness of those tools evaluated. The platforms evaluated were Lego Mindstorms, Arduino, and Micro:bit. The author extended the evaluation to include and assess other tools including programming environments which can be used with the three platforms to provide a more holistic experience with those tools.

 The author uses the term "new paradigm" to explain the curriculum that integrates Papert's constructionism and maker movements while focusing on creativity and 21st-century skills. However, those have already been used in educational contexts for more than 10 years. If this approach is new in Greece, it needs to be addressed in the paper.

 Regarding the evaluation of the robotics tools, there is no explanation of why those three tools were selected. There are more and more robotics tools becoming available every year. Although those are some of the most popular tools available, there are other tools similar to those three selected. For example, VEX IQ, Robotis' OLLO, Sony's KOOV are some of the robotics tools similar to LEGO Mindstorms.

 Although the evaluation is comprehensive and touched upon details, some of the categories used to evaluate the tools are more instructional problems than tool-specific issues. For example, the first category of DIY robotics can be influenced by instructions/lessons. For example, craft materials can be used with LEGO Mindstorms to create maker/DIY projects. Although Mindstorms is too big to use with a wearable type of project, it can create dancing robots or robotics toys with which costumes are created using craft materials. Arduino can be used with a project for self-expression using various shields and kits, such as the ones that are mentioned in the paper. Again, it is more instructional than tool-specific.

 Although each robotics tool has some limitations, which tool to use should be guided by the learning goals and outcomes of a lesson/curriculum. If the learning objective is to introduce an electric circuit, Arduino is better than Mindstorm.

 Another factor that should be taken into account when deciding on learning tools to use is the age/developmental stage of students. In the paper, it is not clear which age group the evaluation has targeted. The evaluation of the tools should include the developmental appropriateness of each tool. Although Arduino is a good tool for DIY type projects, it is not developmentally appropriate for lower elementary school students. Although the paper contains useful information for classroom teachers and educators considering using robotics tool to be integrated into their teaching practices especially focusing on inclusive robotics, instead of starting the paper with the information about how each technology satisfies the criteria emerged from the INBOT project and introducing more options that could make the tool more effective tool in terms of satisfying the criteria, I would suggest organizing the discussion around the criteria and how to make each tool effective to integrate the various pedagogies and approaches INBOT promotes for teachers/educators to use in their classrooms.

 Lastly, additional English edit of the manuscript will be helpful.

Are all factual statements correct and adequately supported by citations?

Partly

Is the review written in accessible language?

Yes

Are the conclusions drawn appropriate in the context of the current research literature?

Yes

Is the topic of the review discussed comprehensively in the context of the current literature?

Partly

Reviewer Expertise:

Educational robotics, STEM education, Computer Science Education, curriculum development, AI literacy.

I confirm that I have read this submission and believe that I have an appropriate level of expertise to confirm that it is of an acceptable scientific standard, however I have significant reservations, as outlined above.

# Response

**Published:** 2021-06-10

**Authors:** Dimitris ALIMISIS

**Affiliations:** EDUMOTIVA, Greece

Thanks for your time to review this paper and for your constructive comments and suggestions.  Below are my responses to your comments/suggestions and the revisions made in the paper.

 Regarding the term " *new paradigm*": I agree with you that constructionism and maker movement have already been used in educational contexts, however the dominant framework seems to be different when it comes to the several curricula provided by the producers of popular commercial robotic kits which certainly influence the use of the kits by teachers and children and robotics education inevitably. A recent review [1] of some typical educational robotics curricula supporting well-known technologies has revealed a chaotic landscape where curricula rarely share common elements and principles. Many of them propose usually a restricted step-by-step guided approach for learners to assembly and program prescribed models of robots. This approach is far away from a constructionist or maker education framework and cannot foster creativity, inventiveness, and curiosity for children [1]. We have criticised somewhere else [2] the deficiencies of the current technocentric [3] paradigm in robotics education and have argued for the need of a paradigm shift [2].  This requires a radical change in the current core concepts and practices of robotics education with the focus being on the transformation of robotics education towards an education inclusive of all children which in this sense represents a new paradigm. I have now explained this issue in two new paragraphs added in page 2 & 3.

 Regarding the selection of the reviewed tools, I have now revised the 1 ^st^ paragraph in p. 6 to better explain  why those three tools were selected.  In short, the three tools are among the most popular, represent quite different approaches within educational robotics and have attracted much attention by the research community.

*“some of the categories used to evaluate the tools are more instructional problems than tool-specific issues”: *I agree that some of the categories used to evaluate the three platforms can be influenced at some extent by the teacher’s instructions. Indeed, an aware teacher may suggest some crafting with Lego Mindstorms to decorate the robot but not to make a robot from scratch. And yes, Arduino, if combined with shields or kits like those I recommend in the paper, can improve its potential for self-expression. However, according to a recent review [1] and our experiences from schools at EU level these practices are not the usual or dominant case in schools and the evaluations in Table 1 are based on the currently dominant use of the tools, not the desired ones. While some of the achievements of a certain tool might be improved at some extent if coupled with an aware teacher and a certain pedagogy, I believe that the philosophy and architecture of each tool usually implies (or promotes) a certain learning pathway for users with consequent benefits or shortcomings like those depicted in Table 1. I have now added a paragraph in page 7 to address this issue.

 Regarding the age appropriateness of the reviewed tools, I have now added in page 6 age groups appropriate for each reviewed tool.

 Regarding the organisation of the discussion: The current structure of the discussion is actually organised around the criteria set; it starts with the evaluation of the three popular platforms to explore how well they satisfy the criteria set which is a necessary step before  going to suggestions for alternative or improved technologies made in the final part of the discussion and based again on the criteria set (pages 8-11).

  

1. Sapounidis T., Alimisis D. (2021) Educational Robotics Curricula: Current Trends and Shortcomings. In: Malvezzi M., Alimisis D., Moro M. (eds) Education in & with Robotics to Foster 21st-Century Skills. EDUROBOTICS 2021. Studies in Computational Intelligence, vol 982. Springer, Cham. https://doi.org/10.1007/978-3-030-77022-8_12 .
2. Alimisis D. (2020) Emerging Pedagogies in Robotics Education: Towards a Paradigm Shift. In: Pons J. (eds) Inclusive Robotics for a Better Society. INBOTS 2018. Biosystems & Biorobotics, vol 25. Springer, Cham. https://doi.org/10.1007/978-3-030-24074-5_22
3. Brennan K. (2015) Beyond technocentrism: Supporting constructionism in the classroom. Constructivist Foundations 10(3): 289–296. http://constructivist.info/10/3/289   

# Peer review: Reviewer response for version 1

**DOI:** 10.21956/openreseurope.14391.r26784

**Published:** 2021-05-11

**Authors:** Sarah Matthews

**Affiliations:** 1 Faculty of Engineering, Architecture and Information Technology, The University of Queensland, Brisbane, Australia

This well-written review addresses a set of questions surrounding commonly-used classroom technologies, namely Mindstorms Lego, Arduino and Micro:bit, as well as programming environments for robotics and other maker platforms. It comparatively discusses existing technologies for their ability to support widescale ‘inclusive’ robotics education in schools. The author’s analysis identifies a set of criteria that should be considered when focusing on the potential of hardware and software to be a ‘creative material’ for students, and to support the development of skills such as critical thinking and problem-solving. This is a clear strength of the paper. The discussion is framed well with respect to the different agendas for which technologies are brought into the classroom e.g., for the learning of ICT, the application of engineering concepts, or to encourage meta-cognitive skills such as problem solving or creativity. The paper also introduces the INBOTS project’s results to date and proposes a hypothetical educational robotics project that makes use of existing technologies to enable children to easily make creative and meaningful interactive robots.

 The topic is timely—insights regarding the nature of and possibilities for technologies to become more than a new content area in educational curricula is a key current issue. This is evidenced by contributions to the recent special issue of International Journal of Child-Computer Interaction (Vol. 29, 2021) on *Computing, Design and Making in Education*.

 Below are recommendations to strengthen and clarify the initial version of the paper.

 The paper employs an important but potentially idiosyncratic concept of inclusiveness, as one that “benefits all children regardless of their future educational or professional orientation”; i.e. ‘inclusive’ appears to refer to treating technology education as a general, rather than a specialist, competency, thereby including all students. This is distinct to more orthodox uses that tie inclusivity to accessibility for children with disabilities, e.g., Bar-El *et al*. (2021) ^1^. An explicit definition of inclusive, or clarification of this distinction for readers might avoid some potential misreadings of the motivation of the project.

 Readers who are looking to apply the criteria on page 4 would benefit from knowing from where each of the questions originated. While most of the questions have motivated or have been the subject of previous studies, two of the criteria are introduced without reference to prior work. An explanatory note detailing their provenance, even if they derive from (non-citable) sources such as personal experience or anecdote, will both add clarity and help readers determine the degree of importance to attach to the individual criteria.

 Table 1 presents an important contribution, providing an overview of existing technologies set against the criteria. But it treats each of the criteria as binary yes/no; in doing so it overstates the case for/against certain technologies. A qualitative, or possibly just a +/- scale would seem to be more accurate, particularly for the more qualitative criteria.

 The recommendation on page 6 to “rethink the role of a technical language… [that] may be strange for young learners”… and to use a more “human” language in coding is one that some other researchers in this space would debate. It might be worth the paper noting in passing, (with Papert), that while it is important to lower the floor to participation, it is also important to do so in ways that do not also lower the ceiling for what children can learn and build with them (Papert, 1980) ^2^. This appears to be where some of the resistance to less technical, more “human” programming languages stems.

 Some additional references to existing research and the issues the paper has identified would better ground the selection of these particular technologies for explicit review:

 Lego Mindstorms: (Castledine, 2011) ^3^ (Üçgül, 2013) ^4^ raise issues that are involved in implementing Mindstorms in a problem solving environment; Arduino: (Agatolio and Moro, 2017 ^5^; Bekker *et al*., 2015 ^6^; Smith *et al*., 2015 ^7^); Micro:bit: (Sentance *et al*., 2017 ^8^; Videnovik *et al*., 2018 ^9^).

 The structure of the “Discussion” is not currently visible in the paper. But there are clearly different sections, and distinct purposes that ought to be structurally differentiated: Establishing criteria, Analysing three technologies against the criteria, Reviewing programming languages and tools, Reviewing maker kits, Reviewing friendly adaptations to Arduino, Proposing a hypothetical project. There is currently not a summary of the review(s) to argue how they collectively answer the second research question; it is answered only implicitly in the positive through a proposal for a hypothetical project. A short summary following the reviews that returns to the criteria would be valuable.

Are all factual statements correct and adequately supported by citations?

Partly

Is the review written in accessible language?

Yes

Are the conclusions drawn appropriate in the context of the current research literature?

Yes

Is the topic of the review discussed comprehensively in the context of the current literature?

Partly

Reviewer Expertise:

Interaction design, educational technologies, creative materials for children, analysis of children's interactions with technologies.

I confirm that I have read this submission and believe that I have an appropriate level of expertise to confirm that it is of an acceptable scientific standard, however I have significant reservations, as outlined above.

## References

[1] : Making the maker movement more inclusive: Lessons learned from a course on accessibility in making. *International Journal of Child-Computer Interaction* .2021;29: 10.1016/j.ijcci.2021.100285 10.1016/j.ijcci.2021.100285

[2] : Mindstorms: Children, Computers, And Powerful Ideas. *Hachette UK* .1980;

[3] : Lego robotics: an authentic problem solving tool?. *in Lee, K.T., King, D., Hudson, P., Chandra, V. (Eds.), Proceedings of the 1st International Conference of STEM in Education 2010* .2011;1-13

[4] : History and Educational Potential of LEGO Mindstorms NXT. *Mersin Üniversitesi Eğitim Fakültesi Dergisi 11* .2013;

[5] : A Workshop to Promote Arduino-Based Robots as Wide Spectrum Learning Support Tools.457: 10.1007/978-3-319-42975-5_11 113-125 10.1007/978-3-319-42975-5_11

[6] : Teaching children digital literacy through design-based learning with digital toolkits in schools. *International Journal of Child-Computer Interaction* .2015;5: 10.1016/j.ijcci.2015.12.001 29-38 10.1016/j.ijcci.2015.12.001

[7] : Design thinking for digital fabrication in education. *International Journal of Child-Computer Interaction* .2015;5: 10.1016/j.ijcci.2015.10.002 20-28 10.1016/j.ijcci.2015.10.002

[8] : Teaching with physical computing devices: the BBC micro:bit initiative. *in: Proceedings of the 12th Workshop on Primary and Secondary Computing Education* .2017; 10.1145/3137065.3137083 87-96 10.1145/3137065.3137083

[9] : The BBC Micro:bit in the International Classroom: Learning Experiences and First Impressions. *in: 2018 17th International Conference on Information Technology Based Higher Education and Training (ITHET)* .2018; 10.1109/ITHET.2018.8424786 1-5 10.1109/ITHET.2018.8424786

# Response

**Published:** 2021-06-10

**Authors:** Dimitris ALIMISIS

**Affiliations:** EDUMOTIVA, Greece

Thanks for your time to review this paper and for your constructive comments and recommendations. Below are my responses to your comments/recommendations and the corresponding revisions made in the paper.

 Regarding the concept of inclusiveness: this paper is based on the framework of the INBOTS project (Inclusive Robotics for a Better Society), and more specifically on WP3 (Promoting Highly Accessible and Multidisciplinary Robotics Education Programs). The concept of inclusiveness in INBOTS relates to the broader social uptake of robotics. In line with this, inclusive robotics education in the paper refers to an education that will make the multiple benefits coming from robotics  education accessible for all the children, demystify robots and familiarise children with them, develop skills for all, and finally promote the future adoption of robots in their work or everyday life.  This concept of inclusiveness comes to challenge the misconception  that fluency with robotic technologies is one more vocational skill relevant only for students who will follow robotics or engineering careers in the future. This misconception, coupled with gender-biased views that robotics is a “male” subject, may result in discouragement or exclusion of students not interested in engineering careers and especially the female ones. Hence, the concept of inclusiveness  in this paper means a robotics education that offers knowledge and skills valuable for every citizen and is clearly distinct to accessibility for children with disabilities. I have now added a paragraph in page 2 to clarify the concept of inclusiveness.

 “ *two of the criteria are introduced without reference to prior work*...” I have now added references to literature and to our experiences from a previous project to help readers know the provenance of the questions (Should technologies prepare students for engineering jobs or develop personal expression and skills? Cost matters) (in page 5 & 6).

 Regarding your suggestion for a qualitative scale in Table 1, I make now use of an ordinal scale consisting of a spectrum of qualitative values for comparing the performance of platforms (excellent, good, average, poor, very poor).

 Regarding your comment on the “ *low floor – high ceiling*” issue in programming tools: “Technical language” and “human language” in the paper refer to the terminology used not only in text coding (e.g. Arduino IDE) but also inside the visual blocks in many programming tools  intended for young learners which in many cases just replicate the terminology used in text coding intended for professionals. Our main concern in this paper is how to make programming work meaningful and accessible for young learners removing barriers imposed by an unnecessarily technical terminology; hence, the focus in this paper is to ensure a low floor for young learners in programming. While I believe the recommendation for making programming meaningful for learners should be  considered also when it comes to “high ceiling”,  I agree with you that efforts to provide a low floor in programming should not undermine a high ceiling (which is important for learners to reach). I find useful to this end the opportunity for young learners to have parallel access to visual blocks and text coding tools. For instance, when learners program their Arduino board using visual blocks in Open Roberta Lab, Arduino C text coding  is automatically generated and appears in a second window. This parallel access helps to familiarise children with C coding and prepare them to jump into a more advanced programming tool when they feel ready to go. I have now added in p. 8 a paragraph addressing your comment.

 “ *Some additional references to existing research and the issues the paper has identified*” have now been added.

 “ *The structure of the “Discussion” is not currently visible in the paper*”: Some subheadings have now been inserted to make the structure visible.

 “ *A short summary following the reviews that returns to the criteria would be valuable*”: I have now added in page 11 a summary of the reviews.
